# Stair use and risk of incident rheumatic and non-rheumatic valvular heart disease: a cohort study from the UK biobank

**DOI:** 10.3389/fcvm.2026.1855909

**Published:** 2026-06-29

**Authors:** Yabing Hou, Huichao Wu, Hongxi Yang, Zuolin Lu

**Affiliations:** 1 Department of Health Data Science, School of Medical Technology, Capital Medical University, Beijing, China; 2 School of Population Medicine and Public Health, Chinese Academy of Medical Sciences & Peking Union Medical College, Beijing, China; 3 Peking Union Medical College Hospital, Chinese Academy of Medical Sciences & Peking Union Medical College, Beijing, China; 4 Department of Bioinformatics, School of Basic Medical Sciences, Tianjin Medical University, Tianjin, China

**Keywords:** non-rheumatic valve disease, population-based study, rheumatic valve disease, stair climbing, valvular heart disease

## Abstract

**Background:**

This study aims to comprehensively investigate the association between stair climbing and risk of incident valvular heart disease (VHD) and its subtypes.

**Methods:**

Participants with available measurements of stair climbing at baseline from 2006 to 2010 were included from the UK Biobank study. Stair climbing data was collected through touchscreen questionnaire. The primary outcome was new-onset VHD, which was defined using ICD-10 code. Incident VHD was followed till March 1st, 2022. Cox models were used to assess the association between stair climbing and VHD.

**Results:**

A total of 488,964 participants (mean age 56.5 years) were included in the present study. During a median follow-up time of 13.0 years, 17,494 (3.6%) participants experienced new-onset VHD. After adjustments, climbing 10–50, 60–100, 110–150 and ≥160 steps of stairs per day were associated with significant reduced VHD risks of 12%, 15%, 22%, and 20%, respectively (*P* for trend <0.0001), compared to not climbing any stairs. For VHD subtypes, individuals who climbed more than 160 steps per day exhibited a lowered risk of rheumatic valve disease (Hazard ratio: 0.79, 95% confidence intervals: 0.66–0.95), non-rheumatic valve disease (0.78, 0.72–0.83), multiple valve disease 0.82, 0.74–0.90), and endocarditis (0.63, 0.48–0.83). Such associations were more evident among participants with lower levels of total physical activity.

**Conclusions:**

In this large population-based cohort study, the number of climbed stairs was independently associated with decreased risk of new-onset VHD. Findings suggest that promoting regular stair climbing could be a potential target for preventive interventions of VHD onset.

## Introduction

Valvular heart disease (VHD), encompassing both rheumatic and non-rheumatic etiologies, poses a significant burden globally (1). VHD was estimated to affect 40.5 million people worldwide in 2019, with an incidence of 2.8 million per year, and accounted for 306,000 global deaths (2). VHD predominantly affects younger age groups, and thus lead to a significant loss of 10.7 million disability-adjusted life-years per year (2), which highlights the importance of exploration into preventive strategies for VHD.

Physical activity stands as a cornerstone in the pursuit of cardiovascular health. Considerable evidence supports the protective role of moderate-to-vigorous intensity physical activity in incident cardiovascular diseases, such as coronary heart disease (CHD) (3) and heart failure (HF) (4), and cardiovascular mortality (5). Among the various forms of physical exertion, stair climbing presents a pragmatic yet often underestimated exercise modality that holds promising implications for cardiometabolic health (6–9). Recently, moderate-to-vigorous intensity physical activity has been linked to a reduced risk of VHD (10), yet specific investigations delving into the relationship between stair climbing, a kind of moderate-to-vigorous intensity activity as well (11), and the incidence of VHD remain undocumented. Stair climbing is an easy and accessible way to incorporate physical activity into daily life. Assessing the potential health impact of stair climbing at a population level is clinically relevant because it can serve as a simple, cost-effective intervention to promote physical activity and to improve cardiovascular health.

The present study aims to elucidate the potential protective effects of regular stair climbing against incident rheumatic and non-rheumatic valvular heart disease. We seek to provide a comprehensive analysis that sheds light on the association of stair climbing with both rheumatic and non-rheumatic diseases. We further evaluate whether the associations are independent of total physical activity.

## Methods

### Study population

Data were obtained from the UK Biobank database. The UK Biobank is a large prospective population-based cohort study which recruited over 500,000 UK general participants aged between 40 and 69 years in 2006∼2010 (12). These participants provided medical history, health behavior, physical measures, and biological samples at the time of enrolment. This study followed the Strengthening the Reporting of Observational Studies in Epidemiology (STROBE) reporting guideline.

**Figure 1:**
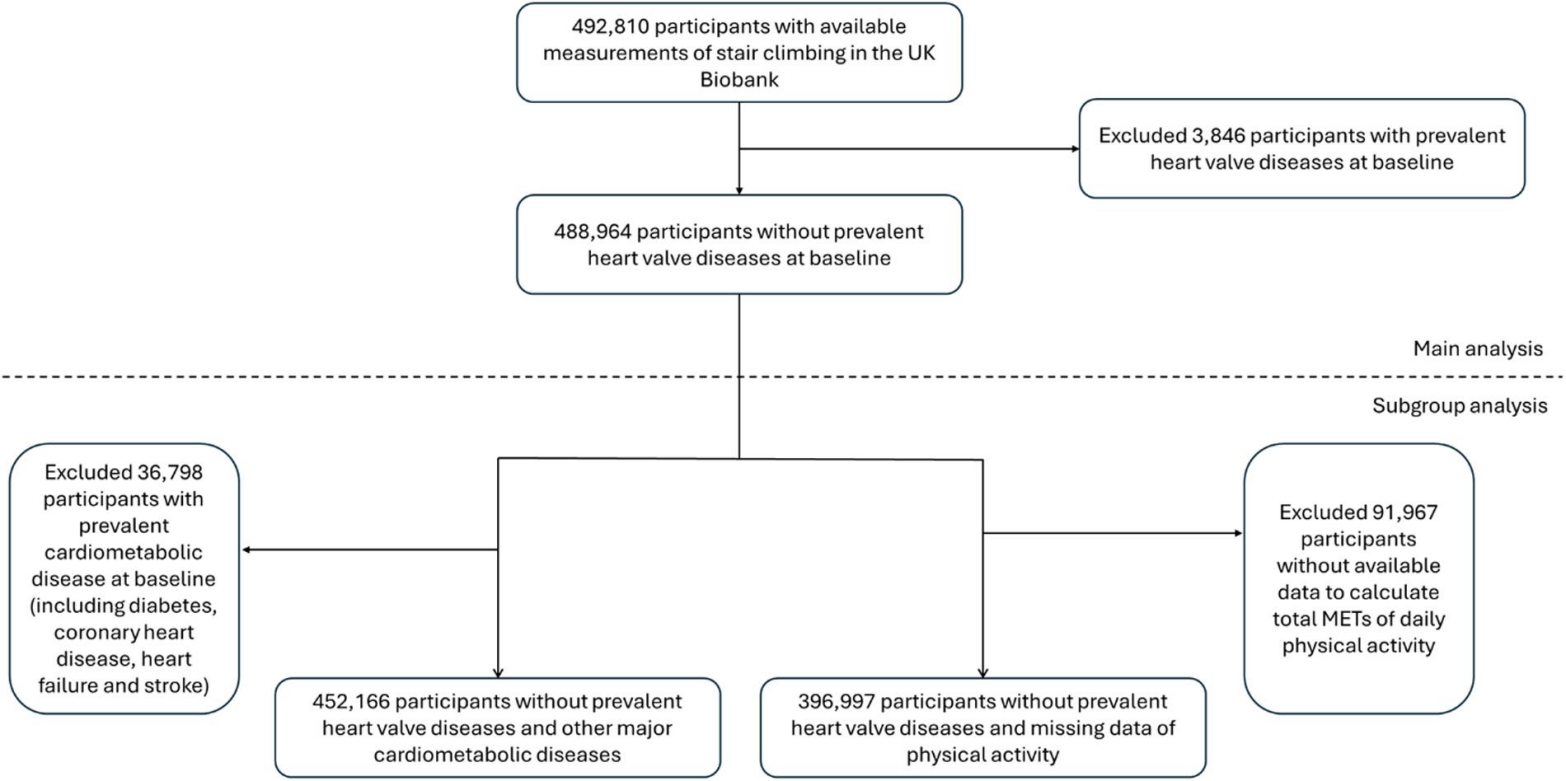
Flowchart of the study population.

In the current study, 492,810 participants with available measurements of stair climbing were considered for inclusion. Further, participants with various prevalent heart valve diseases at baseline were excluded (*N* = 3,846). Finally, there were 488,964 participants included in analysis (Figure 1).

### Assessment of stair climbing

Stair climbing was self-reported by participants by answering the following question (touchscreen questionnaire): ‘At home, during the last 4 weeks, about how many times a DAY do you climb a flight of stairs? (approximately 10 steps)’. Possible answers were none, 1–5 times a day, 6–10 times a day, 11–15 times a day, 16–20 times a day, or more than 20 times a day. For better understanding, we recoded participants into five groups by steps instead of flights as (1) none, (2) 10–50 steps, (3) 60–100 steps, (4) 110–150 steps and (5) ≥ 160 steps. Long-term reliability of stair climbing was validated previously in a subset of UK Biobank participants assessed by a quadratic weighed kappa examination with the coefficient of 0.62 (8). Participants with no stair climbing were set as the reference.

### Assessment of rheumatic and non-rheumatic valvular heart disease

Various VHDs were assessed using the hospital admission, primary care and/or death registry data linked to the UK Biobank (12). Included heart valve diseases and their corresponding International Classification of Diseases (ICD)-10 codes were: I05 (rheumatic mitral valve diseases), I06 (rheumatic aortic valve diseases), I07 (rheumatic tricuspid valve diseases), I08 (multiple valve diseases), I34 (nonrheumatic mitral valve disorders), I35 (nonrheumatic aortic valve disorders), I36 (nonrheumatic tricuspid valve disorders), I37 (pulmonary valve disorders), I38 (endocarditis, valve unspecified), and I39 (endocarditis and heart valve disorders in diseases classified elsewhere). We then regrouped all patients into four kinds of heart valve disease: 1) rheumatic valve disease (I05-07); 2) non-rheumatic valve disease (I34-37); 3) multiple valve disease (I08); and 4) endocarditis (I38 and I39). The follow-up ended on March 1st, 2022. Participants were censored at the end of follow-up, the date of incident heart valve disease, the date of death, or loss to follow-up whichever occurred first.

### Assessment of covariables

Assessment of covariables at baseline were described previously (12). In brief, age, sex, education, employment, alcohol intake status, smoking status, and medication use for blood pressure and/or cholesterol were obtained directly from the touchscreen questionnaires. The Townsend deprivation index was used as a marker of area-based socioeconomic status (13). Blood pressure was automatically measured twice during an interview using an Omron 705 IT electronic blood pressure monitor. Height (in meters) and weight (in kilogram) were measured in the assessment centers, and body mass index (BMI) was calculated as weight in kilograms divided by weight in meters squared. Physical activity was assessed using the International Physical Activity Questionnaire (IPAQ), which asks about the frequency, intensity, duration of walking and other moderate and vigorous activity in the last 4 weeks, and is scored according to the protocol to estimate total metabolic equivalent of tasks (METs). Total METs/week was summed to represent total amount of physical activity. We further defined the moderate physical activity if participants had ≥150 min/week of moderate activity or ≥75 min/week of vigorous activity or ≥150 min/week of moderate and vigorous activity (14). Healthy diet was assessed based on consumption of at least four of seven commonly eaten food groups following recommendations on dietary priorities for cardiometabolic health (15) Prevalent cardiometabolic comorbidities at baseline, including diabetes mellitus [DM; ICD10 codes: E10-14], CHD [ICD-10 codes: I20-22,24,25], stroke (ICD codes: I60-64), and HF [ICD-10 codes: I50] was defined by ICD-10 codes on basis of the hospital admission and/or primary care. All self-reported cases of prevalent cardiometabolic disorders at baseline were set to missing and excluded from the analysis. The UK Biobank Field IDs for each variable were listed in the supplementary materials in Supplementary Table S1.

### Statistical analyses

Multivariable Cox proportional hazards analyses between stair climbing and incident VHD was performed to quantify the associations. In the first model, we adjusted the analyses for age and sex (Model 1). In the second model, we additionally adjusted for education, employment, BMI, Townsend deprivation index, systolic blood pressure, diastolic blood pressure, low-density lipoprotein cholesterol, triglycerides, C-reactive protein, smoking status, alcohol intake status, healthy diet (yes/no), moderate physical activity (yes/no), blood pressure and cholesterol lowering medication (yes/no), and history of DM (yes/no), CHD (yes/no), stroke (yes/no) and HF (yes/no) (Model 2). Age, BMI, systolic/diastolic blood pressure, low-density lipoprotein cholesterol, triglycerides, C-reactive protein, and Townsend deprivation index were modeled as continuous variables. Education level was modeled as a categorical variable (University/college degree or other). The proportional hazards assumption was tested using Schoenfeld residuals and if any violation was detected for any variables, we had used stratified Cox models. To account for the competing risk of death, we also performed sensitivity analyses using the Fine-Gray subdistribution hazards model.

To test the potential effect modifications, we further stratified participants according to sex and total physical activity categories (<500 METs/week, 500–1,500 METs/week, 1,500–2,500 METs/week, 2,500–5,000 METs/week, and >5,000 METs/week), and interactions were further examined.

In sensitivity analyses, we repeated the analyses for individual non-rheumatic heart valve disease including mitral valve disease, aortic valve disease, tricuspid valve disease and pulmonary valve disease, respectively. In addition, participants reported cardiometabolic diseases, including DM, CHD, stroke, and HF, at baseline were excluded to rule out the potential influence by comorbidities (*N* = 452,166). Age stratified analysis were conducted with interaction tests in participants <60 years or ≥60 years. Finally, to minimize the potential influence of reverse causation, we further repeated the main analyses after excluding participants who developed VHD within the first three years of follow-up.

Missing values in covariates were handled using multiple imputation by chained equations (MICE) with 5 imputed datasets, under the missing-at-random assumption. Supplementary Table S2 summarized the proportion of missing data for each covariate in the primary sample, including the number and percentage missing. Statistical significance was considered at two-tailed *P*-value <0.05. In sensitivity analyses, a complete case analysis was carried out (*N* = 280,255). The analyses were done using statistical software R (R 4.0.2; R Foundation for Statistical Computing, Vienna, Austria).

## Results

A total of 488,964 participants (mean age 56.5 ± 8.1 years) at baseline were eligible for the analyses. The baseline characteristics for our study are shown in Table 1. Baseline characteristics among participants with specific VHD subtypes were reported in the supplementary materials in Supplementary Table S3∼S6. The median follow-up period was 13.0 years (interquartile range 12.2–13.7 years) during which 17,494 (3.6%) experienced incident VHD. The incidence rate was 2.9 per 1,000 person years.

**Table 1:** Baseline characteristics.

Characteristic	Overall population
No. of participants	488,964
Male, N (%)	222,866 (45.6)
Age, mean (SD), years	56.5 (8.1)
Ethnicity, N (%)	-
White	442,011 (90.4)
Other	46,953 (9.6)
Body mass index, mean (SD), kg/m2	27.4 (4.8)
Diastolic blood pressure, mean (SD), mmHg	82 (11)
Systolic blood pressure, mean (SD), mmHg	140 (20)
C-reactive protein, mean (SD), mg/L	2.6 (4.4)
Low-density lipoprotein cholesterol, mean (SD), mmol/L	3.6 (0.9)
Triglycerides, mean (SD), mmol/L	1.8 (1.0)
University or college educational level, N (%)	332,160 (67.9)
Current employment, N (%)	-
Worked	283,032 (57.9)
Retired	162,464 (33.2)
Unemployed	37,915 (7.8)
None of the above	5,553 (1.1)
Income, N (%), pounds/year	-
Less than 18,000	111,726 (22.8)
18,000 to 30,999	124,336 (25.4)
31,000 to 51,999	127,506 (26.1)
52,000 to 100,000	99,294 (20.3)
>100,000	26,102 (5.3)
Townsend deprivation index, mean (SD)	−1.3 (3.1)
Moderate activity, yes, N (%)	307,040 (62.8)
Healthy diet, yes, N (%)	98,821 (20.2)
Smoking status, N (%)	**-**
Never	267,700 (54.7)
Previous	169,366 (34.6)
Current	51,898 (10.6)
Alcohol status, N (%)	**-**
Never	21,403 (4.4)
Previous	17,354 (3.5)
Current	450,207 (92.1)
Blood pressure and/or lipid lowering medication, N (%)	128,538 (26.3)
Disease history, N (%)	-
Heart failure	1,976 (0.4)
Diabetes	15,536 (3.2)
Stroke	3,558 (0.7)
Coronary heart disease	21,722 (4.4)
Stair climbing, N (%), steps/day	-
None	44,531 (9.1)
10–50	99,670 (20.4)
60–100	177,568 (36.3)
110–150	90,690 (18.5)
≥160	76,505 (15.6)

### Association between stair climbing and the risk of the development of all kinds of VHDs

 Table 2 outlines the relationship between the number of steps climbed on stairs per day and the risk of new-onset VHD. In the basic model (Model 1), a significant linear association was established between an increased number of climbed stairs and a reduced risk of incident VHD (*P* for trend <0.001). This association was slightly attenuated but remained statistically significant after adjusting for potential confounders (Model 2). When compared to the reference group (no stair climbing), regularly climbing stairs was associated with a lower risk of incident VHD, showing a hazard ratio (HR) of 0.88 [95% confidence interval (CI): 0.82–0.94] for 10–50 steps/day, HR of 0.85 (95% CI: 0.79–0.90) for 60–100 steps/day, HR of 0.78 (95% CI: 0.72–0.84) for 110–150 steps/day, and HR of 0.80 (95% CI: 0.74–0.86) for >150 steps/day. Supplementary Table S7 shows the sex-specific associations of stair climbing with incident VHD. Regular stair climbing was associated with reduced risk of VHD among both men and women, and there no significant sex interaction was observed (P for sex interaction=0.86).

**Table 2:** Associations between stair climbing and risk of incident VHD (combined all kinds of VHDs).

Stair climbing (steps/day)	No. of cases/observations	Hazard ratio (95% confidence intervals)	
		Model 1	Model 2
None	2,234/44,531	1.00 (Reference)	1.00 (Reference)
10–50	3,886/99,670	0.76 (0.72–0.80)	0.88 (0.82–0.94)
60–100	6,152/177,568	0.67 (0.64–0.70)	0.85 (0.79–0.90)
110–150	2,850/90,690	0.60 (0.57–0.64)	0.78 (0.72–0.84)
≥160	2,372/76,505	0.60 (0.56–0.63)	0.80 (0.74–0.86)
*P for trend*	-	<0.0001	<0.0001

Model 1 was adjusted for age and sex (if applicable).

Model 2 was additionally adjusted for ethnicity, education, employment, total family income, Townsend deprivation index, body mass index, low-density lipoprotein cholesterol, triglycerides, C-reactive protein, diastolic blood pressure, systolic blood pressure, medication use of cholesterol and/or blood pressure, smoking status, alcohol intake status, moderate physical activity (yes/no), healthy diet (yea/no), and history of coronary heart disease, stroke, diabetes, and heart failure.

### Association between stair climbing and the risk of incident VHD subtypes

In Table 3, the relationship between stair climbing and various subtypes of incident VHD (Valvular Heart Disease) is depicted, encompassing rheumatic valve disease, non-rheumatic valve disease, multiple valve disease, and endocarditis. Upon thorough adjustments (Model 2), it was found that individuals who climbed more than 160 steps per day exhibited a lowered risk of rheumatic valve disease (HR: 0.79, 95% CI: 0.66–0.95) compared to those who did not climb stairs at all. Furthermore, an increase in the number of climbed stairs showed a significant association with reduced risks of non-rheumatic valve disease, multiple valve disease, and endocarditis. For example, people who climbed more than 160 steps per day had a 22% reduced risk for incident non-rheumatic valve disease (HR: 0.78, 95% CI: 0.72–0.83), a 18% reduced risk for incident multiple valve disease (HR: 0.82, 95% CI: 0.74–0.90), and a 37% reduced risk for incident endocarditis (HR: 0.63, 95% CI: 0.48–0.83).

**Table 3:** Associations between stair climbing and various types of VHD.

Stair climbing (steps/day)	Types of heart valve disease (No. of cases/observations)			
	Rheumatic valve disease (1,914/488,964)	Non-rheumatic valve disease (12,062/488,964)	Multiple valve disease (6,566/488,964)	Endocarditis (746/488,964)
Model 1				
** **None	1.00 (Reference)	1.00 (Reference)	1.00 (Reference)	1.00 (Reference)
** **10–50	0.86 (0.74–1.01)	0.76 (0.71–0.81)	0.78 (0.72–0.85)	0.61 (0.48–0.78)
** **60–100	0.67 (0.57–0.77)	0.67 (0.64–0.71)	0.66 (0.61–0.72)	0.53 (0.42–0.65)
** **110–150	0.65 (0.55–0.77)	0.61 (0.57–0.65)	0.57 (0.52–0.63)	0.45 (0.35–0.58)
** **≥160	0.59 (0.49–0.70)	0.60 (0.56–0.64)	0.61 (0.55–0.66)	0.45 (0.35–0.59)
** **P for trend	<0.0001	<0.0001	<0.0001	<0.0001
Model 2				
** **None	1.00 (Reference)	1.00 (Reference)	1.00 (Reference)	1.00 (Reference)
** **10–50	0.98 (0.84–1.15)	0.86 (0.80–0.91)	0.90 (0.83–0.98)	0.71 (0.56–0.90)
** **60–100	0.85 (0.73–0.99)	0.83 (0.78–0.88)	0.85 (0.78–0.92)	0.69 (0.55–0.86)
** **110–150	0.87 (0.74–1.04)	0.78 (0.73–0.83)	0.76 (0.69–0.83)	0.62 (0.48–0.81)
** **≥160	0.79 (0.66–0.95)	0.78 (0.72–0.83)	0.82 (0.74–0.90)	0.63 (0.48–0.83)
** **P for trend	<0.01	<0.0001	<0.0001	<0.01

Values are shown as hazard ratio (HR) and 95% confidence intervals (95% CI). Model 1 was adjusted for age and sex. Model 2 was additionally adjusted for ethnicity, education, employment, total family income, Townsend deprivation index, body mass index, low-density lipoprotein cholesterol, triglycerides, C-reactive protein, diastolic blood pressure, systolic blood pressure, medication use of cholesterol and/or blood pressure, smoking status, alcohol intake status, moderate physical activity (yes/no), healthy diet (yea/no), and history of coronary heart disease, stroke, diabetes, and heart failure.

Supplementary Table S8 illustrates the association between stair climbing and specific non-rheumatic heart valve diseases, including mitral valve disease, aortic valve disease, tricuspid valve disease, and pulmonary valve disease. Overall, after adjustments for age and sex, a protective role of stair climbing was observed across all subtypes of non-rheumatic heart valve diseases. However, further adjustments for other confounders diluted the associations between stair climbing and tricuspid valve disease as well as pulmonary valve disease. Compared to those who did not climb stairs, participants who climbed more than 160 steps per day exhibited a 21% reduced risk for incident mitral valve disease (HR: 0.79, 95% CI: 0.71–0.88) and a 27% reduced risk for incident aortic valve disease (HR: 0.73, 95% CI: 0.66–0.80). Borderline significance was observed in the associations between climbing more than 160 steps per day and incident tricuspid valve disease (HR: 0.73, 95% CI: 0.52–1.03) as well as pulmonary valve disease (HR: 0.76, 95% CI: 0.56–1.05).

### Effect modification by total physical activity on the associations between stair climbing and incident VHDs

 Table 4 shows the associations of stair climbing with total VHD and its subtypes, categorized by strata of total physical activity groups represented by METs/week. In general, significant protective effect of stair climbing on VHD was observed across all physical activity groups, with the strongest association between stair climbing and VHD was observed among participants with the lowest level of physical activity (<500 METs/week) (P for interaction <0.0001). Similar results were found among VHD subtypes, including rheumatic valve disease, non-rheumatic valve disease and multiple valve disease.

**Table 4:** Associations between stair climbing and various types of VHD among groups of total physical activity.

Stair climbing (steps/day)	Physical activity groups				
	<500 METs/week (*N* = 61,730)	500–1,500 METs/week (*N* = 112,385)	1,500–2,500 METs/week (*N* = 75,389)	2,500–5,000 METs/week (*N* = 87,987)	>5,000 METs/week (*N* = 59,506)
All VHDs					
** **None	1.00 (Reference)	1.00 (Reference)	1.00 (Reference)	1.00 (Reference)	1.00 (Reference)
** **10–50	0.88 (0.78–1.00)	0.85 (0.76–0.95)	0.80 (0.69–0.93)	0.93 (0.81–1.07)	0.83 (0.71–0.98)
** **60–100	0.77 (0.67–0.87)	0.87 (0.78–0.97)	0.83 (0.73–0.95)	0.93 (0.83–1.06)	0.84 (0.73–0.98)
** **110–150	0.63 (0.54–0.74)	0.87 (0.77–0.99)	0.79 (0.68–0.92)	0.83 (0.73–0.95)	0.82 (0.70–0.97)
** **≥160	0.75 (0.62–0.89)	0.84 (0.74–0.96)	0.79 (0.67–0.92)	0.80 (0.70–0.93)	0.87 (0.74–1.01)
P for trend	<0.0001	0.08	0.02	<0.001	0.26
P for interaction	<0.001				
Rheumatic valve disease					
** **None	1.00 (Reference)	1.00 (Reference)	1.00 (Reference)	1.00 (Reference)	1.00 (Reference)
** **10–50	0.90 (0.65–1.26)	0.88 (0.62–1.24)	1.18 (0.71–1.98)	1.18 (0.77–1.83)	1.37 (0.82–2.28)
** **60–100	0.64 (0.45–0.91)	0.94 (0.68–1.30)	1.09 (0.67–1.77)	1.31 (0.88–1.96)	0.86 (0.52–1.42)
** **110–150	0.61 (0.38–0.96)	1.03 (0.72–1.48)	1.02 (0.60–1.73)	1.01 (0.65–1.58)	1.04 (0.62–1.77)
** **≥160	0.62 (0.36–1.05)	0.90 (0.60–1.34)	0.97 (0.55–1.69)	0.67 (0.41–1.10)	1.32 (0.80–2.19)
P for trend	<0.01	0.89	0.56	0.03	0.52
P for interaction	0.08				
Non-rheumatic valve disease					
** **None	1.00 (Reference)	1.00 (Reference)	1.00 (Reference)	1.00 (Reference)	1.00 (Reference)
** **10–50	0.88 (0.76–1.02)	0.85 (0.74–0.98)	0.76 (0.64–0.91)	0.93 (0.79–1.10)	0.82 (0.67–1.00)
** **60–100	0.76 (0.66–0.89)	0.88 (0.77–1.00)	0.80 (0.68–0.93)	0.92 (0.79–1.06)	0.87 (0.72–1.03)
** **110–150	0.70 (0.58–0.84)	0.84 (0.73–0.98)	0.72 (0.61–0.86)	0.85 (0.72–1.00)	0.84 (0.69–1.02)
** **≥160	0.73 (0.59–0.91)	0.87 (0.75–1.02)	0.74 (0.61–0.89)	0.82 (0.69–0.97)	0.85 (0.70–1.03)
P for trend	<0.0001	0.21	<0.01	<0.01	0.29
P for interaction	0.01				
Multiple valve disease					
** **None	1.00 (Reference)	1.00 (Reference)	1.00 (Reference)	1.00 (Reference)	1.00 (Reference)
** **10–50	0.82 (0.68–1.00)	0.92 (0.76–1.11)	0.85 (0.66–1.10)	1.04 (0.84–1.30)	0.83 (0.64–1.07)
** **60–100	0.73 (0.60–0.89)	0.92 (0.77–1.10)	0.92 (0.73–1.15)	1.01 (0.82–1.24)	0.79 (0.62–0.99)
** **110–150	0.46 (0.35–0.60)	0.91 (0.75–1.12)	0.83 (0.64–1.08)	0.85 (0.68–1.07)	0.77 (0.60–1.00)
** **≥160	0.76 (0.58–1.00)	0.82 (0.66–1.03)	0.92 (0.70–1.20)	0.90 (0.71–1.13)	0.84 (0.65–1.07)
P for trend	<0.0001	0.14	0.64	0.05	0.27
P for interaction	<0.01				

Values are shown as hazard ratio (HR) and 95% confidence intervals (95% CI). Model was adjusted for age, sex, ethnicity, education, employment, total family income, Townsend deprivation index, body mass index, low-density lipoprotein cholesterol, triglycerides, C-reactive protein, diastolic blood pressure, systolic blood pressure, medication use of cholesterol and/or blood pressure, smoking status, alcohol intake status, healthy diet (yea/no), total METs per week, and history of coronary heart disease, stroke, diabetes, and heart failure. P for interaction <0.001.

### Sensitivity analyses

The complete case analysis showed consistent results compared to the findings following imputation (Supplementary Table S9). After excluding participants with cardiometabolic diseases at baseline from the analyses, the associations between stair climbing and incident VHD remained statistically significant (Supplementary Table S10). To account for the competing risk of death, we performed sensitivity analyses using the Fine-Gray subdistribution hazards model. Results were consistent with the primary Cox models and are presented in Supplementary Table S11 and Supplementary Table S12. In age-stratified analyses (Supplementary Table S13), the inverse association between stair climbing (≥160 vs. none) and incident VHD was similar in participants <60 years (HR 0.85, 95% CI 0.78–0.94) and ≥60 years (HR 0.73, 95% CI 0.66–0.82), with no significant interaction (P for interaction = 0.13).

## Discussion

In this large prospective cohort study of 488,964 participants, we observed a robust, linear association between stair climbing and reduced risk of developing VHD, and its subtypes, including rheumatic valve disease, non-rheumatic valve disease, multiple valve disease, and endocarditis. Remarkably, such associations were independent of total physical activity, suggesting additional preventive benefits specifically linked to regular stair climbing in averting the onset of VHD. Notably, the protective effect was more pronounced among participants with lower levels of weekly physical activity. This underscores the potential for healthcare professionals in clinical and public health practices to encourage individuals who are unable to meet current physical activity recommendations to incorporate frequent stair use, rather than elevators, into their daily routines, thereby reducing the risk of VHD.

Stair climbing has been linked to a reduced risk of cardiometabolic disorders and mortality in various cohort studies. Evidence from the UK Biobank suggests that climbing stairs is associated with a lower risk of all-cause and cancer mortality (8). In a prospective cohort study of the Harvard Alumni Health Study, stair climbing was related to a reduced risk of heart attack (16). A similar association was found for metabolic syndrome (6). Recently, Wu and colleagues reported that a higher number of climbed stairs was associated with a lower risk of type 2 diabetes, with a significant interaction between stair climbing and genetic predisposition to this condition (9).

Consistent with previous evidence, our findings suggest a protective role of regular stair climbing in the occurrence of VHD. Specifically, climbing 60–100 stairs per day (around 3–5 floors/day) was associated with a 15% lower risk of rheumatic valve disease and a 17% lower risk of non-rheumatic valve disease compared to climbing no stairs. Additionally, a clear dose-response relationship was observed between an increased number of climbed stairs and reduced risks of VHD and all its subtypes. Climbing 160 steps or more per day (≥ 8 floors/day) was associated with a 21% lower risk of incident rheumatic valve disease and a 22% lower risk of non-rheumatic valve disease compared to climbing no stairs. To the best of our knowledge, we are the first to report an inverse association between the number of climbed stairs and incident VHD. The observed association between stair climbing and VHD is biologically plausible. As a form of moderate-to-vigorous intensity activity (11), regular stair climbing benefits the improvement of lipoprotein profiles and decreases body fat (17), which are established risk factors for VHD (18). Hypertension is one of the most important risk factors in VHD development (19). Evidence suggests that leisure-time walking might contribute to lower systolic blood pressure among adults with hypertension (20). Specifically, a previous study showed a significant decreased systolic and diastolic blood pressure after a stair climbing regimen (4 d/wk, climbing 192 steps) among postmenopausal women (21). Interestingly, our findings also suggest that regular stair climbing is associated with a lower risk of non-rheumatic valve disease. Rheumatic valve disease is a condition in which the heart valves have been permanently damaged by rheumatic fever, commonly triggered by a strep infection (22). Though lacking of direct evidence, regular moderate-to-vigorous physical activity was associated with a reduced risk of community-acquired infectious diseases reported by a recent meta-analysis (23). Taken together, our findings suggest a significant association between stair climbing and incident VHD. However, due to the observational design, further clinical trials are warranted to evaluate the potential causal relationship.

### Clinical implications

Overwhelming epidemiological evidence supports the role of physical activity as one of the accessible interventions for improving cardiovascular health and reducing the risks of obesity, diabetes, dyslipidemia, hypertension and coronary heart disease (24–26) Thus, it is not surprising that its beneficial impact extends to preventing VHD as well. However, current epidemiological evidence has concluded an inconsistent association of physical activity with the onset of VHD (10, 27). This inconsistency may be accounted for by the potential detrimental impact of vigorous-intensity physical activity on valve function, as increased hemodynamic loads during exercise could contribute to the elevated risk of VHD (28). Therefore, finding an effective form of exercise, beyond assessing general physical activity, associated with a reduced risk of VHD is of clinical interest in VHD prevention. Our results suggest a significant dose-response relationship between an increased number of climbed stairs and a reduced risk of VHD onset, highlighting the potential recommendation of using stairs in daily life to avert the development of VHD. Importantly, this linear association is more pronounced among participants with a lower amount of total physical activity. Considering that more than a quarter of adults worldwide have insufficient physical activity, our findings suggest that regular stair climbing is a simple and effective physical activity for preventing VHD, especially for those unable to meet current recommendations for total physical activity. Our study holds relevance for public health because interventions aimed at increasing stair climbing appear feasible and effective.

### Strengths and limitations

Main strengths of our study include its large sample size, prospective study design, long follow-up time, and extensive adjustment for a wide range of confounding factors. However, there are several considerations to bear in mind when interpreting our findings. Firstly, the majority of our participants were of European ancestry, potentially limiting the generalizability of our results to other ethnicities. Moreover, the higher disease burden of rheumatic valve disease in developing countries suggests that our findings might underestimate the situation when generalized to these regions. Secondly, the self-reported nature of the number of stair climbing instances introduces the possibility of recall bias, inherent in the use of such questionnaires. Nonetheless, the long-term reliability of stair climbing was previously validated in a subset, showing a quadratic weighted kappa coefficient of 0.62 (8). Thirdly, due to an observational study design, we cannot rule out the possibility of residual or unmeasured confounding. Reverse causation should also be considered when interpreting the association between stair climbing and incident VHD. Participants with subclinical or early-stage VHD may already have reduced exercise tolerance at baseline, leading to lower stair-climbing frequency. To reduce this potential bias, we performed sensitivity analyses excluding participants who developed VHD within the first three years of follow-up, and the results were materially unchanged (Supplementary Table S14). Nevertheless, because subclinical VHD may remain undiagnosed for a prolonged period, residual reverse causation cannot be completely excluded. Therefore, our findings should be interpreted cautiously as observational associations rather than direct evidence that stair climbing causally reduces VHD risk. Fourthly, utilizing ICD-10 codes to define cardiovascular outcomes has limitations, as detection relies on healthcare utilization and may not be perfectly accurate, carrying a potential risk of misclassification (29). Furthermore, our classification of VHD subtypes was primarily based on etiological ICD-10 codes (rheumatic vs. non-rheumatic) rather than specific valvular lesions (e.g., regurgitation vs. stenosis). Future studies using echocardiographic data are needed to assess associations with specific valve pathologies. Fifthly, although we used Cox proportional hazards models, death from other causes may act as a competing risk that could bias cumulative incidence estimates. However, sensitivity analyses using Fine-Gray competing risk models yielded similar findings, suggesting that our results are robust to this potential bias.

## Conclusion

The study revealed a clear dose-response relationship, illustrating that as the number of stairs climbed increased, the risk of VHD and its subtypes decreased accordingly. An especially noteworthy discovery was the more evident protective impact of stair climbing among individuals with lower levels of weekly physical activity. Our findings present the potential for promoting the integration of stair climbing into daily routines as an effective and accessible strategy in both clinical and public health settings. Encouraging individuals to choose stairs over elevators could significantly contribute to reducing the incidence of VHD. Future studies are warranted to test the relationship between stair climbing and incident VHD among developing countries and to further evaluate the potential causality.

## Data Availability

The datasets presented in this article are not readily available because. The data that support the findings of this study are available from UK Biobank (https://www.ukbiobank.ac.uk/), but restrictions apply to the availability of these data, which were used under license for the current study, and so are not publicly available. Data are however available from the authors upon reasonable request and with permission of UK Biobank. Requests to access the datasets should be directed to UK Biobank, ukbiobank@ukbiobank.ac.uk.
