# Supplementary material for: Stair use and risk of incident rheumatic and non-rheumatic valvular heart disease: a cohort study from the UK biobank
Source: Front Cardiovasc Med. 2026 Jun 29;13:1855909. doi: 10.3389/fcvm.2026.1855909 (PMC13357226; doi:10.3389/fcvm.2026.1855909)
Supplement: Supplementary file 1 [file Datasheet1.docx]

Online-Only Supplements for the Manuscript:

Title: Stair use and risk of incident rheumatic and non-rheumatic valvular heart disease: a cohort study from the UK Biobank

Table S1. UK Biobank Field IDs of assessed variables

| Variable | UK Biobank Field ID(s) |
| --- | --- |
| Stair climbing | 359 |
| Body mass index | 21001 |
| Systolic blood pressure | 4080 |
| Diastolic blood pressure | 4079 |
| Townsend deprivation index | 189 |
| Ethnicity | 21000 |
| C-reactive protein | 30710 |
| Low-density lipoprotein cholesterol | 30780 |
| Triglycerides | 30870 |
| Education | 6138 |
| Employment | 6142 |
| Income | 738 |
| Medication | 6177, 6153 |
| Healthy Diet | 1309, 1319, 1289, 1299, 1438, 1448, 1458, 1468, 1329, 1339, 6144, 1408, 1418, 2654, 1349, 1369, 1379, 1389, 3680, 1428 |
| Smoking status | 20116 |
| Alcohol intake status | 1558 |
| ICD-10 codes for definitions of outcomes | 41270, 41280 |
| Date of death | 40000 |
| Physical activity (IPAQ) | 884-891, 904-911 |

Table S2. Proportion of missing data for each covariate in the primary sample (N=488,964)

| **Variables name** | **Number of missing (%)** |
| --- | --- |
| Family income | 69,826 (14.3) |
| Townsend deprivation index | 602 (0.1) |
| Physical activity | 91,967 (18.8) |
| Dietary quality | 23,099 (4.7) |
| Body mass index | 3,000 (0.6) |
| Smoking status | 2,847 (0.6) |
| Alcohol intake status | 587 (0.1) |
| Diastolic blood pressure | 33,469 (6.8) |
| Systolic blood pressure | 33,482 (6.8) |
| C-reactive protein | 32,907 (6.7) |
| Low-density lipoprotein cholesterol | 32,780 (6.7) |
| Triglyceride | 32,283 (6.6) |
| History of heart failure | 96 (0) |
| History of coronary heart disease | 3,801 (0.8) |
| History of stroke | 4,338 (0.9) |
| History of diabetes | 11,128 (2.3) |

Table S3. Baseline characteristics among participants with and without rheumatic valve disease

|  | No rheumatic valve disease | Incident rheumatic valve disease | P values |
| --- | --- | --- | --- |
| No. of participants | 487,050 | 1,914 |  |
| Male, N (%) | 222015 (45.6) | 851 (44.5) | 0.337 |
| Age, mean (SD), years | 56.53 (8.10) | 56.56 (8.03) | 0.859 |
| Ethnicity, N (%) | 46748 (9.6) | 205 (10.7) | 0.107 |
| Body mass index, mean (SD), kg/m2 | 27.44 (4.80) | 27.42 (4.72) | 0.883 |
| Diastolic blood pressure, mean (SD), mmHg | 139.75 (19.70) | 139.45 (19.33) | 0.507 |
| Systolic blood pressure, mean (SD), mmHg | 82.22 (10.71) | 82.54 (10.65) | 0.189 |
| C-reactive protein, mean (SD), mg/L | 2.60 (4.36) | 2.53 (4.04) | 0.449 |
| Low-density lipoprotein cholesterol, mean (SD), mmol/L | 3.56 (0.87) | 3.56 (0.86) | 0.875 |
| Triglycerides, mean (SD), mmol/L | 1.75 (1.03) | 1.77 (1.03) | 0.407 |
| University or college educational level, N (%) | 330888 (67.9) | 1272 (66.5) | 0.174 |
| Current employment, N (%) |  |  | 0.402 |
| Worked | 281897 (57.9) | 1135 (59.3) |  |
| Retired | 161836 (33.2) | 628 (32.8) |  |
| Unemployed | 37783 (7.8) | 132 (6.9) |  |
| None of the above | 5534 (1.1) | 19 (1.0) |  |
| Income, N (%), pounds/year |  |  | <0.001 |
| Less than 18,000 | 110957 (22.8) | 690 (36.1) |  |
| 18,000 to 30,999 | 124161 (25.5) | 540 (28.2) |  |
| 31,000 to 51,999 | 126803 (26.0) | 408 (21.3) |  |
| 52,000 to 100,000 | 99257 (20.4) | 227 (11.9) |  |
| >100,000 | 25872 (5.3) | 49 (2.6) |  |
| Townsend deprivation index, mean (SD) | -1.32 (3.08) | -0.95 (3.24) | <0.001 |
| Moderate activity, yes, N (%) | 305834 (62.8) | 1160 (60.6) | 0.051 |
| Healthy diet, yes, N (%) | 98611 (20.2) | 359 (18.8) | 0.112 |
| Smoking status, N (%) |  |  | 0.525 |
| Never | 266604 (54.7) | 1071 (56.0) |  |
| Previous | 168725 (34.6) | 650 (34.0) |  |
| Current | 51721 (10.6) | 193 (10.1) |  |
| Alcohol status, N (%) |  |  | <0.001 |
| Never | 21291 (4.4) | 127 (6.6) |  |
| Previous | 17256 (3.5) | 99 (5.2) |  |
| Current | 448503 (92.1) | 1688 (88.2) |  |
| Blood pressure and/or lipid lowering medication, N (%) | 127940 (26.3) | 818 (42.7) | <0.001 |
| Disease history, N (%) |  |  |  |
| Heart failure | 1931 (0.4) | 49 (2.6) | <0.001 |
| Diabetes | 15566 (3.2) | 186 (9.7) | <0.001 |
| Stroke | 3513 (0.7) | 37 (1.9) | <0.001 |
| Coronary heart disease | 21475 (4.4) | 270 (14.1) | <0.001 |
| Stair climbing, N (%), steps/day |  |  | <0.001 |
| None | 44294 (9.1) | 237 (12.4) |  |
| 10-50 | 99207 (20.4) | 463 (24.2) |  |
| 60-100 | 176923 (36.3) | 645 (33.7) |  |
| 110-150 | 90367 (18.6) | 323 (16.9) |  |
| ≥160 | 76259 (15.7) | 246 (12.9) |  |

Table S4. Baseline characteristics among participants with and without non-rheumatic valve disease

|  | No non-rheumatic valve disease | Incident non-rheumatic valve disease | P values |
| --- | --- | --- | --- |
| No. of participants | 476,902 | 12,062 |  |
| Male, N (%) | 217351 (45.6) | 5515 (45.7) | 0.757 |
| Age, mean (SD), years | 56.52 (8.09) | 56.70 (8.14) | 0.019 |
| Ethnicity, N (%) | 45824 (9.6) | 1129 (9.4) | 0.368 |
| Body mass index, mean (SD), kg/m2 | 27.44 (4.80) | 27.37 (4.76) | 0.129 |
| Diastolic blood pressure, mean (SD), mmHg | 139.75 (19.69) | 139.89 (19.77) | 0.419 |
| Systolic blood pressure, mean (SD), mmHg | 82.22 (10.71) | 82.20 (10.73) | 0.898 |
| C-reactive protein, mean (SD), mg/L | 2.60 (4.36) | 2.58 (4.37) | 0.585 |
| Low-density lipoprotein cholesterol, mean (SD), mmol/L | 3.56 (0.87) | 3.55 (0.87) | 0.306 |
| Triglycerides, mean (SD), mmol/L | 1.75 (1.03) | 1.75 (1.04) | 0.751 |
| University or college educational level, N (%) | 323985 (67.9) | 8175 (67.8) | 0.717 |
| Current employment, N (%) |  |  | 0.427 |
| Worked | 276076 (57.9) | 6956 (57.7) |  |
| Retired | 158411 (33.2) | 4053 (33.6) |  |
| Unemployed | 37011 (7.8) | 904 (7.5) |  |
| None of the above | 5404 (1.1) | 149 (1.2) |  |
| Income, N (%), pounds/year |  |  | <0.001 |
| Less than 18,000 | 107648 (22.6) | 3999 (33.2) |  |
| 18,000 to 30,999 | 121259 (25.4) | 3442 (28.5) |  |
| 31,000 to 51,999 | 124578 (26.1) | 2633 (21.8) |  |
| 52,000 to 100,000 | 97852 (20.5) | 1632 (13.5) |  |
| >100,000 | 25565 (5.4) | 356 (3.0) |  |
| Townsend deprivation index, mean (SD) | -1.32 (3.08) | -1.14 (3.20) | <0.001 |
| Moderate activity, yes, N (%) | 299631 (62.8) | 7363 (61.0) | <0.001 |
| Healthy diet, yes, N (%) | 96474 (20.2) | 2496 (20.7) | 0.215 |
| Smoking status, N (%) |  |  | 0.709 |
| Never | 261109 (54.8) | 6566 (54.4) |  |
| Previous | 165154 (34.6) | 4221 (35.0) |  |
| Current | 50639 (10.6) | 1275 (10.6) |  |
| Alcohol status, N (%) |  |  | <0.001 |
| Never | 20834 (4.4) | 584 (4.8) |  |
| Previous | 16757 (3.5) | 598 (5.0) |  |
| Current | 439311 (92.1) | 10880 (90.2) |  |
| Blood pressure and/or lipid lowering medication, N (%) | 123699 (25.9) | 5059 (41.9) | <0.001 |
| Disease history, N (%) |  |  |  |
| Heart failure | 1704 (0.4) | 276 (2.3) | <0.001 |
| Diabetes | 14681 (3.1) | 1071 (8.9) | <0.001 |
| Stroke | 3362 (0.7) | 188 (1.6) | <0.001 |
| Coronary heart disease | 19995 (4.2) | 1750 (14.5) | <0.001 |
| Stair climbing, N (%), steps/day |  |  | <0.001 |
| None | 42990 (9.0) | 1541 (12.8) |  |
| 10-50 | 97018 (20.3) | 2652 (22.0) |  |
| 60-100 | 173310 (36.3) | 4258 (35.3) |  |
| 110-150 | 88721 (18.6) | 1969 (16.3) |  |
| ≥160 | 74863 (15.7) | 1642 (13.6) |  |

Table S5. Baseline characteristics among participants with and without multiple valve disease

|  | No multiple valve disease | Incident multiple valve disease | P values |
| --- | --- | --- | --- |
| No. of participants | 482,398 | 6,566 |  |
| Male, N (%) | 219851 (45.6) | 3015 (45.9) | 0.587 |
| Age, mean (SD), years | 56.53 (8.10) | 56.66 (8.12) | 0.172 |
| Ethnicity, N (%) | 46351 (9.6) | 602 (9.2) | 0.238 |
| Body mass index, mean (SD), kg/m2 | 27.44 (4.80) | 27.51 (4.85) | 0.208 |
| Diastolic blood pressure, mean (SD), mmHg | 139.74 (19.70) | 140.19 (19.45) | 0.068 |
| Systolic blood pressure, mean (SD), mmHg | 82.21 (10.70) | 82.57 (10.79) | 0.008 |
| C-reactive protein, mean (SD), mg/L | 2.60 (4.36) | 2.65 (4.42) | 0.369 |
| Low-density lipoprotein cholesterol, mean (SD), mmol/L | 3.56 (0.87) | 3.53 (0.87) | 0.019 |
| Triglycerides, mean (SD), mmol/L | 1.75 (1.03) | 1.77 (1.04) | 0.103 |
| University or college educational level, N (%) | 327659 (67.9) | 4501 (68.6) | 0.285 |
| Current employment, N (%) |  |  | 0.747 |
| Worked | 279261 (57.9) | 3771 (57.4) |  |
| Retired | 160265 (33.2) | 2199 (33.5) |  |
| Unemployed | 37389 (7.8) | 526 (8.0) |  |
| None of the above | 5483 (1.1) | 70 (1.1) |  |
| Income, N (%), pounds/year |  |  | <0.001 |
| Less than 18,000 | 109354 (22.7) | 2293 (34.9) |  |
| 18,000 to 30,999 | 122777 (25.5) | 1924 (29.3) |  |
| 31,000 to 51,999 | 125864 (26.1) | 1347 (20.5) |  |
| 52,000 to 100,000 | 98670 (20.5) | 814 (12.4) |  |
| >100,000 | 25733 (5.3) | 188 (2.9) |  |
| Townsend deprivation index, mean (SD) | -1.32 (3.08) | -1.00 (3.26) | <0.001 |
| Moderate activity, yes, N (%) | 302957 (62.8) | 4037 (61.5) | 0.029 |
| Healthy diet, yes, N (%) | 97623 (20.2) | 1347 (20.5) | 0.589 |
| Smoking status, N (%) |  |  | 0.320 |
| Never | 264141 (54.8) | 3534 (53.8) |  |
| Previous | 167054 (34.6) | 2321 (35.3) |  |
| Current | 51203 (10.6) | 711 (10.8) |  |
| Alcohol status, N (%) |  |  | <0.001 |
| Never | 21073 (4.4) | 345 (5.3) |  |
| Previous | 17004 (3.5) | 351 (5.3) |  |
| Current | 444321 (92.1) | 5870 (89.4) |  |
| Blood pressure and/or lipid lowering medication, N (%) | 125905 (26.1) | 2853 (43.5) | <0.001 |
| Disease history, N (%) |  |  |  |
| Heart failure | 1798 (0.4) | 182 (2.8) | <0.001 |
| Diabetes | 15089 (3.1) | 663 (10.1) | <0.001 |
| Stroke | 3428 (0.7) | 122 (1.9) | <0.001 |
| Coronary heart disease | 20648 (4.3) | 1097 (16.7) | <0.001 |
| Stair climbing, N (%), steps/day |  |  | <0.001 |
| None | 43688 (9.1) | 843 (12.8) |  |
| 10-50 | 98169 (20.4) | 1501 (22.9) |  |
| 60-100 | 175267 (36.3) | 2301 (35.0) |  |
| 110-150 | 89676 (18.6) | 1014 (15.4) |  |
| ≥160 | 75598 (15.7) | 907 (13.8) |  |

Table S6. Baseline characteristics among participants with and without endocarditis

|  | No endocarditis | Incident endocarditis | P values |
| --- | --- | --- | --- |
| No. of participants | 488,218 | 746 |  |
| Male, N (%) | 222514 (45.6) | 352 (47.2) | 0.398 |
| Age, mean (SD), years | 56.53 (8.10) | 56.66 (8.04) | 0.658 |
| Ethnicity, N (%) | 46884 (9.6) | 69 (9.2) | 0.791 |
| Body mass index, mean (SD), kg/m2 | 27.44 (4.80) | 27.66 (4.96) | 0.201 |
| Diastolic blood pressure, mean (SD), mmHg | 139.75 (19.70) | 140.02 (18.41) | 0.710 |
| Systolic blood pressure, mean (SD), mmHg | 82.22 (10.71) | 82.28 (10.70) | 0.871 |
| C-reactive protein, mean (SD), mg/L | 2.60 (4.36) | 2.52 (4.38) | 0.598 |
| Low-density lipoprotein cholesterol, mean (SD), mmol/L | 3.56 (0.87) | 3.52 (0.86) | 0.243 |
| Triglycerides, mean (SD), mmol/L | 1.75 (1.03) | 1.81 (1.13) | 0.124 |
| University or college educational level, N (%) | 331637 (67.9) | 523 (70.1) | 0.217 |
| Current employment, N (%) |  |  | 0.183 |
| Worked | 282627 (57.9) | 405 (54.3) |  |
| Retired | 162197 (33.2) | 267 (35.8) |  |
| Unemployed | 37848 (7.8) | 67 (9.0) |  |
| None of the above | 5546 (1.1) | 7 (0.9) |  |
| Income, N (%), pounds/year |  |  | <0.001 |
| Less than 18,000 | 111355 (22.8) | 292 (39.1) |  |
| 18,000 to 30,999 | 124480 (25.5) | 221 (29.6) |  |
| 31,000 to 51,999 | 127085 (26.0) | 126 (16.9) |  |
| 52,000 to 100,000 | 99390 (20.4) | 94 (12.6) |  |
| >100,000 | 25908 (5.3) | 13 (1.7) |  |
| Townsend deprivation index, mean (SD) | -1.32 (3.08) | -1.01 (3.30) | 0.006 |
| Moderate activity, yes, N (%) | 306553 (62.8) | 441 (59.1) | 0.042 |
| Healthy diet, yes, N (%) | 98831 (20.2) | 139 (18.6) | 0.294 |
| Smoking status, N (%) |  |  | 0.743 |
| Never | 267260 (54.7) | 415 (55.6) |  |
| Previous | 169117 (34.6) | 258 (34.6) |  |
| Current | 51841 (10.6) | 73 (9.8) |  |
| Alcohol status, N (%) |  |  | <0.001 |
| Never | 21374 (4.4) | 44 (5.9) |  |
| Previous | 17308 (3.5) | 47 (6.3) |  |
| Current | 449536 (92.1) | 655 (87.8) |  |
| Blood pressure and/or lipid lowering medication, N (%) | 128448 (26.3) | 310 (41.6) | <0.001 |
| Disease history, N (%) |  |  |  |
| Heart failure | 1956 (0.4) | 24 (3.2) | <0.001 |
| Diabetes | 15678 (3.2) | 74 (9.9) | <0.001 |
| Stroke | 3541 (0.7) | 9 (1.2) | 0.183 |
| Coronary heart disease | 21616 (4.4) | 129 (17.3) | <0.001 |
| Stair climbing, N (%), steps/day |  |  | <0.001 |
| None | 44412 (9.1) | 119 (16.0) |  |
| 10-50 | 99505 (20.4) | 165 (22.1) |  |
| 60-100 | 177313 (36.3) | 255 (34.2) |  |
| 110-150 | 90578 (18.6) | 112 (15.0) |  |
| ≥160 | 76410 (15.7) | 95 (12.7) |  |

Table S7. Association between stair climbing and risk of incident heart valve disease among men and women

| Stair climbing (steps/day) | No. of cases/observations | | Hazard ratio (95% confidence intervals) | |
| --- | --- | --- | --- | --- |
|  |  |  | Model 1 | Model 2 |
| *Men* | | | | |
| None | | 1255/23,067 | 1.00 (Reference) | 1.00 (Reference) |
| 10-50 | | 2102/51,926 | 0.79 (0.73-0.86) | 0.90 (0.83-0.97) |
| 60-100 | | 3307/93,107 | 0.70 (0.65-0.76) | 0.87 (0.81-0.94) |
| 110-150 | | 1519/48,106 | 0.65 (0.60-0.70) | 0.84 (0.77-0.91) |
| ≥160 | | 1306/40,403 | 0.61 (0.56-0.67) | 0.80 (0.74-0.88) |
| P for trend | | - | <0.0001 | <0.0001 |
| *Women* | | | | |
| None | | 979/19,230 | 1.00 (Reference) | 1.00 (Reference) |
| 10-50 | | 1784/43,858 | 0.74 (0.69-0.79) | 0.84 (0.79-0.90) |
| 60-100 | | 2845/78,309 | 0.64 (0.60-0.69) | 0.80 (0.75-0.86) |
| 110-150 | | 1331/39,734 | 0.57 (0.53-0.62) | 0.74 (0.69-0.80) |
| ≥160 | | 1066/33,730 | 0.58 (0.54-0.63) | 0.77 (0.71-0.83) |
| P for trend | | - | <0.0001 | <0.0001 |

Model 1 was adjusted for baseline age.

Model 2 was additionally adjusted for ethnicity, education, employment, total family income, Townsend deprivation index, body mass index, low-density lipoprotein cholesterol, triglycerides, C-reactive protein, diastolic blood pressure, systolic blood pressure, medication use of cholesterol and/or blood pressure, smoking status, alcohol intake status, moderate physical activity (yes/no), healthy diet (yea/no), and history of and history of coronary heart disease, stroke, diabetes, and heart failure. P for sex interaction = 0.86.

Table S8. Association between stair climbing and specific non-rheumatic heart valve disease

| Stair climbing (steps/day) | Specific non-rheumatic heart valve disease (No. of cases/observations) | | | |
| --- | --- | --- | --- | --- |
|  | Mitral valve disease  (5,410/483,554) | Aortic valve disease  (6,450/482,514) | Tricuspid valve disease  (472/488,492) | Pulmonary valve disease  (746/488,218) |
| Model 1 | | | | |
| None | 1.00 (Reference) | 1.00 (Reference) | 1.00 (Reference) | 1.00 (Reference) |
| 10-50 | 0.74 (0.67-0.81) | 0.74 (0.68-0.80) | 0.65 (0.48-0.89) | 0.89 (0.67-1.16) |
| 60-100 | 0.69 (0.63-0.76) | 0.62 (0.58-0.68) | 0.58 (0.44-0.76) | 0.72 (0.56-0.93) |
| 110-150 | 0.65 (0.59-0.72) | 0.56 (0.51-0.61) | 0.46 (0.33-0.64) | 0.69 (0.52-0.92) |
| ≥160 | 0.63 (0.56-0.70) | 0.55 (0.50-0.60) | 0.52 (0.37-0.73) | 0.58 (0.43-0.80) |
| *P for trend* | <0.0001 | <0.0001 | <0.0001 | <0.0001 |
| Model 2 | | | | |
| None | 1.00 (Reference) | 1.00 (Reference) | 1.00 (Reference) | 1.00 (Reference) |
| 10-50 | 0.82 (0.75-0.90) | 0.84 (0.78-0.92) | 0.74 (0.54-1.00) | 1.01 (0.77-1.33) |
| 60-100 | 0.83 (0.76-0.91) | 0.78 (0.72-0.85) | 0.75 (0.57-1.00) | 0.90 (0.70-1.17) |
| 110-150 | 0.81 (0.73-0.90) | 0.73 (0.66-0.80) | 0.63 (0.45-0.88) | 0.90 (0.67-1.20) |
| ≥160 | 0.79 (0.71-0.88) | 0.73 (0.66-0.80) | 0.73 (0.52-1.03) | 0.76 (0.56-1.05) |
| P for trend | <0.001 | <0.0001 | 0.06 | 0.048 |

Values are shown as hazard ratio (HR) and 95% confidence intervals (95% CI). Model 1 was adjusted for age and sex. Model 2 was additionally adjusted for ethnicity, education, employment, total family income, Townsend deprivation index, body mass index, low-density lipoprotein cholesterol, triglycerides, C-reactive protein, diastolic blood pressure, systolic blood pressure, medication use of cholesterol and/or blood pressure, smoking status, alcohol intake status, moderate physical activity (yes/no), healthy diet (yea/no), and history of coronary heart disease, stroke, diabetes, and heart failure.

Table S9. Complete case analysis

| Stair climbing (steps/day) | Subgroup analysis |
| --- | --- |
|  | Complete case analysis  (N=280,255) |
| Full adjusted model | |
| None | 1.00 (Reference) |
| 10-50 | 0.87 (0.81-0.93) |
| 60-100 | 0.83 (0.78-0.89) |
| 110-150 | 0.76 (0.71-0.82) |
| ≥160 | 0.77 (0.72-0.84) |
| P for trend | <0.0001 |

Values are shown as hazard ratio (HR) and 95% confidence intervals (95% CI). Model was adjusted for age, sex, ethnicity, education, employment, total family income, Townsend deprivation index, body mass index, low-density lipoprotein cholesterol, triglycerides, C-reactive protein, diastolic blood pressure, systolic blood pressure, medication use of cholesterol and/or blood pressure, smoking status, alcohol intake status, healthy diet (yea/no), moderate physical activity (yes/no), and history of coronary heart disease, stroke, diabetes, and heart failure.

| Stair climbing (steps/day) | Subgroup analysis |
| --- | --- |
|  | Without cardiometabolic diseases  (N=452,166) |
| None | 1.00 (Reference) |
| 10-50 | 0.86 (0.81-0.92) |
| 60-100 | 0.84 (0.79-0.88) |
| 110-150 | 0.77 (0.72-0.82) |
| ≥160 | 0.79 (0.74-0.85) |
| P for trend | <0.0001 |

Table S10. Sensitivity analysis: Association between stair climbing and heart valve disease among participants without major cardiometabolic diseases at baseline

Values are shown as hazard ratio (HR) and 95% confidence intervals (95% CI). Model was adjusted for age, sex, ethnicity, education, employment, total family income, Townsend deprivation index, body mass index, low-density lipoprotein cholesterol, triglycerides, C-reactive protein, diastolic blood pressure, systolic blood pressure, medication use of cholesterol and/or blood pressure, smoking status, alcohol intake status, healthy diet (yea/no), and moderate physical activity (yes/no).

Table S11. Associations between stair climbing and risk of incident VHD (combined all kinds of VHDs), accounting for potential competing risk of death

| Stair climbing (steps/day) | No. of cases/observations | Hazard ratio (95% confidence intervals) | |
| --- | --- | --- | --- |
|  |  | Model 1 | Model 2 |
| None | 2,234/44,531 | 1.00 (Reference) | 1.00 (Reference) |
| 10-50 | 3,886/99,670 | 0.76 (0.72-0.80) | 0.88 (0.82-0.94) |
| 60-100 | 6,152/177,568 | 0.67 (0.64-0.70) | 0.85 (0.79-0.90) |
| 110-150 | 2,850/90,690 | 0.60 (0.57-0.64) | 0.78 (0.72-0.84) |
| ≥160 | 2,372/76,505 | 0.60 (0.56-0.63) | 0.80 (0.74-0.86) |
| *P for trend* | - | <0.0001 | <0.0001 |

Model 1 was adjusted for age and sex (if applicable).

Model 2 was additionally adjusted for ethnicity, education, employment, total family income, Townsend deprivation index, body mass index, low-density lipoprotein cholesterol, triglycerides, C-reactive protein, diastolic blood pressure, systolic blood pressure, medication use of cholesterol and/or blood pressure, smoking status, alcohol intake status, moderate physical activity (yes/no), healthy diet (yea/no), and history of and history of coronary heart disease, stroke, diabetes, and heart failure.

Table S12. Associations between stair climbing and various types of VHD, accounting for potential competing risk of death

| Stair climbing (steps/day) | Types of heart valve disease (No. of cases/observations) | | | |
| --- | --- | --- | --- | --- |
|  | Rheumatic valve disease  (1,914/488,964) | Non-rheumatic valve disease  (12,062/488,964) | Multiple valve disease  (6,566/488,964) | Endocarditis  (746/488,964) |
| Model 1 |  |  |  |  |
| None | 1.00 (Reference) | 1.00 (Reference) | 1.00 (Reference) | 1.00 (Reference) |
| 10-50 | 0.86 (0.74-1.01) | 0.76 (0.71-0.81) | 0.78 (0.72-0.85) | 0.61 (0.48-0.78) |
| 60-100 | 0.67 (0.57-0.77) | 0.67 (0.64-0.71) | 0.66 (0.61-0.72) | 0.53 (0.42-0.65) |
| 110-150 | 0.65 (0.55-0.77) | 0.61 (0.57-0.65) | 0.57 (0.52-0.63) | 0.45 (0.35-0.58) |
| ≥160 | 0.59 (0.49-0.70) | 0.60 (0.56-0.64) | 0.61 (0.55-0.66) | 0.45 (0.35-0.59) |
| P for trend | <0.0001 | <0.0001 | <0.0001 | <0.0001 |
| Model 2 |  |  |  |  |
| None | 1.00 (Reference) | 1.00 (Reference) | 1.00 (Reference) | 1.00 (Reference) |
| 10-50 | 0.97 (0.81-1.16) | 0.86 (0.80-0.91) | 0.90 (0.83-0.98) | 0.71 (0.56-0.90) |
| 60-100 | 0.81 (0.68-0.96) | 0.83 (0.78-0.88) | 0.85 (0.78-0.92) | 0.69 (0.55-0.86) |
| 110-150 | 0.81 (0.67-0.99) | 0.78 (0.73-0.83) | 0.76 (0.69-0.83) | 0.62 (0.48-0.81) |
| ≥160 | 0.74 (0.61-0.91) | 0.78 (0.72-0.83) | 0.82 (0.74-0.90) | 0.63 (0.48-0.83) |
| P for trend | <0.01 | <0.0001 | <0.0001 | <0.01 |

Values are shown as hazard ratio (HR) and 95% confidence intervals (95% CI). Model 1 was adjusted for age and sex. Model 2 was additionally adjusted for ethnicity, education, employment, total family income, Townsend deprivation index, body mass index, low-density lipoprotein cholesterol, triglycerides, C-reactive protein, diastolic blood pressure, systolic blood pressure, medication use of cholesterol and/or blood pressure, smoking status, alcohol intake status, moderate physical activity (yes/no), healthy diet (yea/no), and history of and history of coronary heart disease, stroke, diabetes, and heart failure.

Table S13. Associations between stair climbing and risk of incident VHD (combined all kinds of VHDs), stratified by baseline age

| Stair climbing (steps/day) | Age groups | |
| --- | --- | --- |
|  | <60 years old  (N=277,377) | >= 60 years old  (N=211,587) |
| None | 1.00 (Reference) | 1.00 (Reference) |
| 10-50 | 0.93 (0.85-1.01) | 0.87 (0.79-0.97) |
| 60-100 | 0.88 (0.81-0.96) | 0.81 (0.74-0.89) |
| 110-150 | 0.83 (0.75-0.91) | 0.76 (0.68-0.84) |
| ≥160 | 0.85 (0.78-0.94) | 0.73 (0.66-0.82) |
| *P for trend* | <0.0001 | <0.0001 |
| *P for interaction* | 0.13 | |

Model was adjusted for age, sex, ethnicity, education, employment, total family income, Townsend deprivation index, body mass index, low-density lipoprotein cholesterol, triglycerides, C-reactive protein, diastolic blood pressure, systolic blood pressure, medication use of cholesterol and/or blood pressure, smoking status, alcohol intake status, moderate physical activity (yes/no), healthy diet (yea/no), and history of and history of coronary heart disease, stroke, diabetes, and heart failure.

Table S14. Associations between stair climbing and risk of incident VHD among participants with follow-up time more than 3 years

| Stair climbing (steps/day) | Subgroup analysis |
| --- | --- |
|  | Follow-up time more than 3 years |
| Full adjusted model | |
| None | 1.00 (Reference) |
| 10-50 | 0.85 (0.79-0.92) |
| 60-100 | 0.83 (0.77-0.89) |
| 110-150 | 0.76 (0.70-0.82) |
| ≥160 | 0.79 (0.73-0.85) |
| P for trend | <0.0001 |

Model was adjusted for age, sex, ethnicity, education, employment, total family income, Townsend deprivation index, body mass index, low-density lipoprotein cholesterol, triglycerides, C-reactive protein, diastolic blood pressure, systolic blood pressure, medication use of cholesterol and/or blood pressure, smoking status, alcohol intake status, moderate physical activity (yes/no), healthy diet (yea/no), and history of and history of coronary heart disease, stroke, diabetes, and heart failure.
